# Ethical leadership and organisational citizenship behaviour: the mediating role of employee commitment and the moderating role of Islamic work ethic

**DOI:** 10.3389/fpsyg.2026.1847356

**Published:** 2026-06-29

**Authors:** Fahad Saeed Al-Subaey

**Affiliations:** Police College, Qatar Police Academy, Doha, Qatar

**Keywords:** employee commitment, ethical leadership, Islamic work ethic, moderated mediation, organisational behaviour, organisational citizenship behaviour

## Abstract

**Introduction:**

Organisational citizenship behaviour (OCB) is important for organisational effectiveness because employees frequently contribute beyond formal role requirements. Although ethical leadership has been associated with citizenship behaviour, less is known about the attitudinal mechanism through which this relationship operates and the conditions under which it becomes stronger.

**Methods:**

This study examines whether employee commitment mediates the relationship between ethical leadership and OCB and whether Islamic Work Ethic (IWE) strengthens this indirect relationship. Data were collected from 350 employees across public-sector and large organisational settings in Qatar and analysed using partial least squares structural equation modelling (PLS-SEM) with bootstrapping.

**Results:**

Ethical leadership was positively associated with employee commitment, which in turn predicted both citizenship behaviour directed toward individuals and toward the organisation. Islamic Work Ethic strengthened the ethical leadership–commitment relationship and the corresponding indirect relationships with both forms of citizenship behaviour.

**Discussion:**

The study extends ethical leadership and OCB research within an Arab and Islamic organisational context. The findings clarify the commitment-based mechanism linking ethical leadership to discretionary behaviour and identify Islamic Work Ethic as a follower-value condition that strengthens this process.

## 1 Introduction

Employees often contribute to organisational life in ways that extend beyond formal role requirements. They assist colleagues, protect service quality, share knowledge, and take initiative when routine systems fall short. Such extra-role contributions are commonly conceptualised as organisational citizenship behaviour (OCB), originally developed as a construct to capture discretionary behaviours that support organisational functioning but are not formally required or directly rewarded (Smith et al., 1983). OCB has long been recognised as important for organisational effectiveness, coordination, and social functioning in organisations (Organ and Ryan, 1995; Podsakoff et al., 2000; Podsakoff and MacKenzie, 2014; Organ, 2018).

Among the leadership approaches examined in this regard, ethical leadership has attracted sustained scholarly attention. Ethical leadership refers to the demonstration of normatively appropriate conduct through personal actions and interpersonal relationships, as well as the promotion of such conduct through communication, reinforcement, and decision-making (Brown et al., 2005; Ng and Feldman, 2015). Ethical leaders are typically viewed as salient role models who signal fairness, integrity, and appropriate conduct in the workplace (Brown et al., 2005; Brown and Treviño, 2006; Mayer et al., 2009). Meta-analytic evidence indicates that ethical leadership is positively associated with a broad range of follower attitudes and behaviours. At the same time, growing scholarship emphasises the need to better understand the mediating processes through which ethical leadership influences employee behaviour and the boundary conditions that shape those processes (Walumbwa et al., 2011; Sun et al., 2024).

In response to these concerns, this study examines whether ethical leadership is associated with organisational citizenship behaviour through employee commitment and whether this indirect relationship depends on employees’ Islamic Work Ethic (IWE). Specifically, we develop and test a first-stage moderated mediation model in which ethical leadership is associated with employee commitment, employee commitment is associated with citizenship behaviour directed toward individuals and toward the organisation, and Islamic Work Ethic strengthens the ethical leadership–commitment relationship. Yet the relationship between ethical leadership and OCB is unlikely to be adequately captured by a simple direct-effect model. Prior and more recent scholarship suggests that understanding this relationship requires closer attention to both the mediating processes through which ethical leadership influences employee behaviour and the boundary conditions that shape those processes (Walumbwa et al., 2011; Sun et al., 2024; Al Halbusi et al., 2023; Aunin et al., 2024; Taamneh et al., 2024). This calls for greater theoretical precision regarding how ethical leadership becomes associated with discretionary employee contribution.

Although previous research has consistently reported positive associations between ethical leadership and organisational citizenship behaviour, three important limitations remain. First, much of the literature has focused primarily on direct relationships, offering less clarity regarding the specific attitudinal mechanisms through which ethical leadership becomes associated with discretionary employee contribution (Bedi et al., 2016; Peng and Kim, 2020; Sun et al., 2024). Second, while some studies acknowledge that leadership effects may vary across followers, less attention has been devoted to follower-level ethical value orientations as conditions shaping how ethical leadership is interpreted and internalised (van Gils et al., 2015; Udin et al., 2022). Third, prior research has often treated organisational citizenship behaviour as a unitary construct, providing less precision regarding whether leadership processes operate similarly across citizenship directed toward individuals and toward the organisation. The present study addresses these gaps by developing a moderated mediation model that positions employee commitment as the central attitudinal mechanism linking ethical leadership to OCBI and OCBO, while Islamic Work Ethic is examined as a follower-level value orientation strengthening this process.

At the same time, scholarship on citizenship behaviour has become more nuanced. Although OCB has traditionally been conceptualised as discretionary and beneficial, subsequent work suggests that extra-role behaviour may also entail personal costs, become socially expected, or reflect normative pressures surrounding what it means to be a “good” employee (Bolino et al., 2010; Bolino and Grant, 2016; Organ, 2018; Yildiz et al., 2023). This does not imply that any single study can determine whether citizenship behaviour is wholly voluntary or pressured. It does, however, reinforce the need for more precise explanatory models that clarify the attitudinal process through which leadership becomes associated with citizenship behaviour.

In response to this need, we propose employee commitment as a key mechanism linking ethical leadership to OCB. A commitment-based account is theoretically grounded because ethical leadership should strengthen employees’ psychological attachment to the organisation, while job attitudes are robust predictors of citizenship behaviour and related forms of work performance (Cropanzano and Mitchell, 2005; Organ and Ryan, 1995; Riketta, 2002; Nguyen et al., 2024). From this perspective, ethical leadership is associated with discretionary contribution not primarily because employees simply mirror leader behaviour, but because leader conduct fosters a stronger attitudinal bond with the organisation that makes extra-role contribution more likely.

We further argue that this indirect process is likely to depend on employees’ value orientation. Leadership effects do not unfold in a vacuum; rather, they depend partly on how followers interpret leader behaviour and the extent to which leader conduct resonates with followers’ own normative frameworks (Edwards and Cable, 2009; van Gils et al., 2015; Udin et al., 2022). In the present study, we examine Islamic Work Ethic (IWE) as a follower-level boundary condition that may strengthen the relationship between ethical leadership and employee commitment (Udin et al., 2022). In this way, the study moves beyond a simple main-effect model and considers when ethical leadership is more strongly associated with discretionary employee contribution.

The study is particularly relevant in the Qatari context, where public-sector organisations operate within institutional environments shaped by strong ethical, social, and religious value systems. As Qatar continues to invest in leadership development, organisational effectiveness, and public-sector modernisation, understanding how ethical leadership influences discretionary employee behaviour becomes increasingly important. Examining Islamic Work Ethic within this setting therefore provides insight into how value-based leadership processes operate in Arab and Islamic organisational contexts. To improve conceptual precision, we distinguish between citizenship behaviour directed toward individuals (OCBI) and citizenship behaviour directed toward the organisation (OCBO) (Lee and Allen, 2002). This distinction matters because helping colleagues and supporting the organisation, although related, represent different forms of extra-role behaviour. Examining both forms allows a more fine-grained account of how ethical leadership and employee commitment relate to different targets of citizenship behaviour.

The theoretical contribution of this study does not rest on the general proposition that ethical leadership is positively associated with organisational citizenship behaviour, which has already received substantial empirical attention. Rather, the study contributes by clarifying the mechanism and condition through which this relationship operates. First, it positions employee commitment as the proximal attitudinal mechanism linking ethical leadership to citizenship behaviour, shifting attention from direct behavioural influence to internalised organisational attachment. Second, it introduces Islamic Work Ethic as a follower-level value orientation that strengthens the ethical leadership–commitment relationship, thereby demonstrating that leadership effects depend partly on whether followers interpret ethical leadership cues as value-consistent and normatively meaningful. Third, by distinguishing between OCBI and OCBO, the study provides a more differentiated explanation of how the commitment-based pathway operates across different targets of discretionary behaviour.

Accordingly, we test a moderated mediation model in which employee commitment mediates the relationship between ethical leadership and both OCBI and OCBO, while Islamic Work Ethic moderates the relationship between ethical leadership and employee commitment.

The remainder of the paper develops the theoretical framework and hypotheses, describes the research design and analytical approach, reports the results, and discusses the theoretical and practical implications of the findings.

## 2 Theory and hypotheses development

Drawing on ethical leadership research, social exchange theory, and follower value-congruence arguments, we develop a first-stage moderated mediation model in which ethical leadership is associated with organisational citizenship behaviour through employee commitment, and this indirect relationship is strengthened by Islamic Work Ethic. The model is grounded in the view that ethical leadership provides salient cues about fairness, integrity, and appropriate conduct, while also shaping employees’ relationship with the organisation (Brown et al., 2005; Brown and Treviño, 2006). From this perspective, ethical leadership should be associated with stronger employee commitment, which in turn should relate positively to citizenship behaviour directed toward individuals (OCBI) and toward the organisation (OCBO). At the same time, because leadership effects depend partly on how followers interpret and respond to leader behaviour, the strength of this attitudinal pathway is expected to vary according to employees’ ethical value orientation. This framing is consistent with prior work suggesting that ethical leadership effects are better explained through intervening attitudinal and relational processes than through simple direct-effect models, and that these effects may vary across follower characteristics (Bedi et al., 2016; Peng and Kim, 2020; van Gils et al., 2015; Makumbe, 2025).

### 2.1 Ethical leadership and employee commitment

Ethical leadership refers to the demonstration of normatively appropriate conduct through personal actions and interpersonal relationships, and the promotion of such conduct through communication, reinforcement, and decision-making (Brown et al., 2005). Ethical leaders are salient sources of moral and behavioural cues for employees because they signal consistency between espoused values and enacted conduct (Brown and Treviño, 2006). When leaders are perceived as fair, trustworthy, and principled, employees are more likely to view the organisation as credible and worthy of organisational attachment. From a social exchange perspective, such leader behaviour can strengthen employees’ felt obligation and attachment to the organisation (Blau, 1964; Cropanzano and Mitchell, 2005). More broadly, ethical leadership research suggests that leadership effects frequently unfold through intervening psychological and relational states rather than through direct behavioural transmission alone (Bedi et al., 2016; Peng and Kim, 2020; Aunin et al., 2024). Nevertheless, prior research has devoted comparatively less attention to whether employee commitment functions as the central attitudinal pathway linking ethical leadership to discretionary forms of employee contribution. Accordingly, ethical leadership should be positively associated with employee commitment.

*H1*: Ethical leadership is positively related to employee commitment.

### 2.2 Employee commitment and organisational citizenship behaviour

Organisational citizenship behaviour refers to discretionary behaviour that supports organisational functioning beyond formal role requirements (Organ, 2018). A useful distinction differentiates citizenship behaviour directed toward individuals (OCBI), such as helping and supporting co-workers, from citizenship behaviour directed toward the organisation (OCBO), such as protecting organisational interests and supporting the broader functioning of the organisation (Williams and Anderson, 1991; Lee and Allen, 2002).

This distinction is theoretically important because different forms of citizenship may be directed toward different targets even when they stem from related motivational states. Despite this distinction, prior ethical leadership research has often treated organisational citizenship behaviour as a unitary construct, offering less precision regarding whether attitudinal mechanisms operate similarly across OCBI and OCBO. Employee commitment is especially relevant in this context because it reflects attachment to and identification with the organisation, which should increase willingness to contribute beyond minimum role requirements (Nabhan and Munajat, 2023). Meta-analytic evidence indicates that attitudinal organisational commitment is positively associated with performance-relevant outcomes, while broader OCB research shows that attitudinal states are among the most robust predictors of citizenship behaviour (Meyer et al., 2002; Organ and Ryan, 1995; Riketta, 2002; García-Cruz and Valle-Cabrera, 2025). Accordingly, employees with stronger commitment should be more likely to engage in both OCBI and OCBO.

*H2a*: Employee commitment is positively related to citizenship behaviour directed toward individuals (OCBI).

*H2b*: Employee commitment is positively related to citizenship behaviour directed toward the organisation (OCBO).

### 2.3 Employee commitment as a mediator

We propose that employee commitment mediates the relationship between ethical leadership and organisational citizenship behaviour. Rather than assuming that ethical leadership directly elicits discretionary contribution, the present model argues that ethical leadership first shapes employees’ psychological attachment to the organisation, and that this attachment then increases the likelihood of extra-role contribution. This logic is consistent with research showing that ethical leadership operates through mediating pathways and that its relationship with citizenship behaviour is often explained through intervening employee states rather than through uniform direct effects (Bedi et al., 2016; Peng and Kim, 2020; Sun et al., 2024). A commitment-based pathway is especially plausible because committed employees are more willing to invest effort, cooperate, and contribute beyond formal role requirements (Cropanzano and Mitchell, 2005; Meyer et al., 2002). Conceptually, this approach shifts attention from ethical leadership as a direct behavioural trigger to employee commitment as the proximal attitudinal mechanism underlying discretionary contribution. In this sense, employee commitment provides a theoretically focused explanation of how ethical leadership becomes associated with both citizenship directed toward individuals and citizenship directed toward the organisation.

*H3a*: Employee commitment mediates the relationship between ethical leadership and citizenship behaviour directed toward individuals (OCBI).

*H3b*: Employee commitment mediates the relationship between ethical leadership and citizenship behaviour directed toward the organisation (OCBO).

### 2.4 Islamic work ethic as a moderator of the ethical leadership–commitment relationship

In this study, Islamic Work Ethic is treated as a follower-level ethical value orientation that shapes how employees interpret leader behaviour. More specifically, it is conceptualised as an interpretive lens through which employees evaluate the ethical coherence and value relevance of leadership signals (Ali, 1992; Ali and Al-Owaihan, 2008). This positioning is consistent with broader work on value congruence, which shows that employees’ value frameworks shape how they interpret and respond to organisational cues (Kristof, 1996; Edwards and Cable, 2009), and with ethical leadership research showing that follower moral orientations can strengthen or weaken leadership effects (van Gils et al., 2015). However, comparatively less research has examined follower-level ethical value orientations as interpretive mechanisms shaping how employees internalise ethical leadership cues into organisational attachment. Within this framework, employees with stronger Islamic Work Ethic should be more likely to recognise ethical leadership as normatively meaningful and value-consistent, thereby strengthening the positive relationship between ethical leadership and employee commitment (Udin, 2025). Although Islamic Work Ethic is contextually specific, its role in the present model is theoretically consistent with the broader proposition that follower value orientations shape the extent to which leadership cues are translated into work attitudes.

*H4*: Islamic Work Ethic positively moderates the relationship between ethical leadership and employee commitment, such that the relationship is stronger when Islamic Work Ethic is higher.

### 2.5 Moderated mediation: conditional indirect effects

Integrating the preceding arguments, we propose a first-stage moderated mediation model in which ethical leadership is associated with employee commitment, employee commitment is associated with OCBI and OCBO, and Islamic Work Ethic strengthens the relationship between ethical leadership and employee commitment. If ethical leadership is more strongly associated with commitment among employees who hold a stronger Islamic Work Ethic, then the indirect relationship between ethical leadership and citizenship behaviour through commitment should also be stronger at higher levels of Islamic Work Ethic. This conditional indirect argument is consistent with growing evidence that ethical leadership effects are process-based (Peng and Kim, 2020; Sun et al., 2024), mechanism-driven, and contingent on follower interpretations and value orientations (Edwards and Cable, 2009; van Gils et al., 2015).

*H5a*: The indirect effect of ethical leadership on citizenship behaviour directed toward individuals (OCBI) via employee commitment is stronger when Islamic Work Ethic is higher.

*H5b*: The indirect effect of ethical leadership on citizenship behaviour directed toward the organisation (OCBO) via employee commitment is stronger when Islamic Work Ethic is higher.

## 3 Research methods

### 3.1 Instrument development

Data were collected using a two-part structured questionnaire. The first section captured respondents’ demographic characteristics, including gender, age, educational level, work experience, and managerial status. The second section assessed the study’s focal latent constructs: ethical leadership, Islamic Work Ethic, employee commitment, and organisational citizenship behaviour. All focal constructs were measured using established multi-item scales to enhance comparability with prior organisational behaviour research and to reduce idiosyncratic measurement error (Brown et al., 2005; Mowday et al., 1979; Organ, 2018).

Consistent with prior work, organisational citizenship behaviour was operationalised as behavioural frequency rather than attitudinal endorsement. Ethical leadership, Islamic Work Ethic, and employee commitment were measured using a 7-point agreement format ranging from 1 (strongly disagree) to 7 (strongly agree). By contrast, organisational citizenship behaviour was measured using a 7-point frequency scale ranging from 1 (never) to 7 (always), with respondents indicating how often they engaged in each citizenship behaviour. This separation between agreement-based and behaviour-frequency formats helps reduce conceptual conflation between values, attitudes, and behaviour, and is consistent with the behavioural status of OCB in the literature (Lee and Allen, 2002; Organ, 2018). Negatively worded items were reverse-coded before analysis.

Ethical leadership was measured using the Ethical Leadership Scale developed by Brown et al. (2005). The scale captures leader role modelling, fairness, and ethical norm-setting, and remains one of the most widely used measures of ethical leadership in organisational research. Islamic Work Ethic was measured using a 17-item scale adapted from Ali (1988), which operationalises work as a moral and social responsibility emphasising effort, honesty, justice, and sincerity of intention. Employee commitment was measured using a 15-item scale from the Organisational Commitment Questionnaire tradition developed by Mowday et al. (1979), capturing identification with, involvement in, and loyalty to the organisation.

Organisational citizenship behaviour was measured using Lee and Allen’s (2002) two-target OCB scale, distinguishing behaviours directed toward individuals (OCBI; 8 items) and behaviours directed toward the organisation (OCBO; 8 items). This distinction improves construct precision by separating discretionary helping directed at co-workers from discretionary contribution directed at the organisation more broadly. In line with the study’s theoretical positioning, OCB is treated as extra-role behaviour beyond formally prescribed job requirements, while the study does not directly measure perceived pressure or compulsory citizenship.

The questionnaire was translated into Arabic using a forward-translation and blind back-translation procedure, and discrepancies were reconciled to improve semantic and conceptual equivalence across language versions (Brislin, 1970; Beaton et al., 2000).

### 3.2 Research design and data collection

The study adopted a quantitative survey design using a structured questionnaire administered to employees across multiple departments in Qatar. Data were collected from employees across public-sector and large organisational settings in Qatar through surveys distributed both in person across multiple departments and electronically via email. The questionnaires were distributed through departmental administrative coordination and professional access channels following institutional approval from the relevant authorised organisational authorities. Participation was voluntary, respondents completed the surveys individually, and completed questionnaires were returned confidentially to the research team without supervisory involvement. Given the organisational setting and access constraints, a non-probability convenience-access sampling approach was used. In organisational survey research, such designs are common when probability sampling is impractical, provided that procedures are transparent and field access is carefully managed (Hulland et al., 2018).

Participation was strictly voluntary, and supervisors were not involved in monitoring participation or collecting responses. Eligibility was limited to current employees who were able to complete the questionnaire independently. A total of 350 usable responses were retained for analysis. This sample size was adequate for estimating the specified model, which included multiple latent constructs, first-stage moderation, and indirect effects (Kock and Hadaya, 2018).

The hypothesised relationships were examined using partial least squares structural equation modelling (PLS-SEM) in SmartPLS 4. PLS-SEM was selected because the study examines a first-stage moderated mediation model involving multiple reflective latent constructs, an interaction term, and bootstrapped indirect and conditional indirect effects. Methodological guidance suggests that PLS-SEM is appropriate when the research objective is to estimate complex explanatory models, assess explained variance, and examine indirect and interaction effects among latent constructs (Hair et al., 2019; Sarstedt et al., 2017; Henseler and Fassott, 2010). The aim of the analysis was not primarily to reproduce the observed covariance matrix or to establish exact global model fit, which is more closely associated with covariance-based SEM (CB-SEM). Rather, the objective was to examine whether the proposed theoretical pattern of relationships explains meaningful variance in employee commitment and citizenship behaviour and whether the hypothesised indirect and conditional indirect effects are supported. This distinction is consistent with Reinartz et al. (2009), who differentiate CB-SEM’s emphasis on covariance reproduction from variance-based SEM’s emphasis on prediction-oriented explanation. PLS-SEM is also suitable for modelling latent-variable interaction effects, particularly where the research model includes moderation among latent constructs (Chin et al., 2003; Henseler and Fassott, 2010). In addition, the use of bootstrapping is appropriate for assessing indirect and conditional indirect effects because such effects often have non-normal sampling distributions (Zhao et al., 2010; Hayes, 2022). At the same time, we recognise the continuing methodological debate surrounding PLS-SEM and therefore interpret the findings conservatively as theory-consistent associations rather than definitive causal estimates (Rönkkö and Evermann, 2013; Henseler et al., 2014).

Bootstrapping with 5,000 resamples was used to assess the significance of direct, indirect, and conditional indirect relationships. This approach is appropriate for evaluating mediation and moderation effects in structural models because it does not rely on normality assumptions for the sampling distribution of indirect effects.

### 3.3 Sample profile

Although the study did not employ probability sampling, the sample included employees from multiple departments, hierarchical levels, and organisational settings within Qatar. The demographic and organisational diversity of the respondents therefore provides reasonable representation of organisational perspectives within the study context and supports the suitability of the sample for examining the proposed relationships within the study context. Table 1 presents the demographic profile of the sample. The final sample comprised 350 employees. Most respondents were male (66.3%), and the largest age group was 30–50 years (56.8%), followed by 51–65 years (26.3%) and below 30 years (16.9%). In terms of education, 70.0% held a Bachelor’s degree, 18.6% a Master’s degree, and 11.4% a doctorate. Work experience was concentrated in the 5–10 year category (34.3%) and the more than 10 years category (31.4%). Most respondents were non-managerial employees (62.8%), while the remainder held supervisory, managerial, or senior leadership roles. Overall, the sample reflects employees with substantial organisational experience across a range of hierarchical levels.

**Table 1:** Sample characteristics (*N* = 350).

Variable	Category	*N*	%
Gender	Male	232	66.3
	Female	118	33.7
Age (years)	Below 30	59	16.9
	30–50	199	56.8
	51–65	92	26.3
Education	Bachelor’s degree	245	70
	Master’s degree	65	18.6
	Doctorate	40	11.4
Work experience	Less than 1 year	30	8.6
	1–3 years	43	12.3
	3–5 years	47	13.4
	5–10 years	120	34.3
	More than 10 years	110	31.4
Managerial status	Non-managerial employee	220	62.8
	Supervisor/team lead	50	14.3
	Manager/head of unit	60	17.2
	Director/senior leader	20	5.7

### 3.4 Ethics and consent

Participation was voluntary. Before completing the questionnaire, respondents received an information statement explaining the study’s purpose, the absence of right or wrong answers, and their right to decline participation or withdraw at any time without consequence. Informed consent was obtained from all participants. The study was conducted as a minimal-risk, anonymous organisational survey in accordance with the applicable institutional ethics and research-governance procedures and applicable data-protection requirements.

No identifying information was collected, and responses were treated as anonymous and confidential. Permission to conduct the study and distribute the surveys was obtained from the relevant authorised unit within the participating organisations. Data were stored securely, accessed only by the research team, and used solely for research purposes.

### 3.5 Common method Bias

Because all focal variables were collected from the same respondents using a single questionnaire, common method variance was treated as a potential concern. Common method bias may arise when covariance among constructs is partly attributable to the measurement method rather than to the theoretical relationships among the constructs (Podsakoff et al., 2003; Podsakoff et al., 2012). We therefore applied both procedural and statistical remedies, as recommended in prior methodological research. At the design stage, the questionnaire separated demographic questions from substantive measures, used established multi-item scales, employed different response formats for attitudinal constructs and behavioural-frequency measures, assured respondents of anonymity, and emphasised that there were no right or wrong answers. Such procedural remedies are recommended to reduce evaluation apprehension, consistency motifs, social desirability, and respondents’ tendency to rely on a single response pattern across constructs (Podsakoff et al., 2003; Podsakoff et al., 2012).

At the statistical level, Harman’s single-factor test was used only as a supplementary diagnostic rather than as the sole basis for assessing common method bias. This is because prior methodological work has cautioned that *post hoc* statistical tests, including Harman’s single-factor test, have limited ability to detect or rule out common method variance on their own (Podsakoff et al., 2003; Richardson et al., 2009). The first factor explained 32.516% of the variance, below the conventional 50% threshold. In addition, full-collinearity diagnostics were inspected in the PLS-SEM model. The VIF values ranged from 1.252 to 2.172, which are below the commonly recommended threshold of 3.3 for detecting potential common method bias using the full-collinearity approach in PLS-SEM (Kock, 2015). These diagnostics reduce concern that common method variance substantially inflated the observed relationships. Nevertheless, because the study remains cross-sectional and single-source, common method bias cannot be ruled out completely. We therefore treat CMV as a residual limitation and interpret the findings with appropriate caution (Podsakoff et al., 2012; Richardson et al., 2009) (see Table 2).

**Table 2:** Common method bias assessment using the full collinearity (inner VIF) approach.

Construct	EL	IWE	EC	OCBI	OCBO
VIF	1.882	2.172	1.252	1.684	1.761

Values represent the maximum inner VIF obtained when each construct was specified as an endogenous variable in the full-collinearity test.

## 4 Data analysis strategy

The hypothesised relationships among the latent constructs were examined using structural equation modelling. Because the model includes multiple reflective constructs, first-stage moderation, mediation, and conditional indirect relationships, PLS-SEM was employed as the primary estimation strategy. In comparison with covariance-based SEM, variance-based SEM can be advantageous when estimating relatively complex explanatory models that emphasise latent variable prediction and indirect-effect structure (Reinartz et al., 2009; Hair et al., 2022). The analytical strategy was therefore aligned with the study’s theoretical objective of evaluating whether the proposed pattern of direct, indirect, and conditional indirect relationships is consistent with the data.

Following recommended SEM practice (Hair et al., 2022), the analysis proceeded in two stages: assessment of the measurement model and assessment of the structural model. The measurement model evaluation focused on indicator reliability, internal consistency, convergent validity, and discriminant validity. The structural model evaluation then examined the hypothesised direct, indirect, and moderated relationships using bootstrapped path estimates. In line with methodological cautions surrounding PLS-SEM, the resulting estimates are interpreted as theory-consistent associations rather than precise causal effects (Rönkkö and Evermann, 2013).

### 4.1 Measurement model

Following established PLS-SEM procedures, the reliability and validity of the measurement model were assessed before evaluating the structural relationships among the constructs (Hair et al., 2022). The measurement model was assessed to establish the reliability and validity of the focal constructs. As shown in Table 3, the retained indicators loaded satisfactorily on their intended constructs. Five indicators (EL3, EC5, IWE9, IWE14, and OCB15) were removed because their outer loadings were below 0.50. Consistent with reflective measurement-model guidance, these indicators were removed only after confirming that the retained items continued to represent the substantive domain of the constructs adequately rather than materially narrowing construct meaning (Hulland, 1999).

**Table 3:** Measurement model assessment.

Construct	Item	Loadings	Cronbach’s alpha	Composite reliability	Average variance extracted (AVE)
Ethical Leadership	EL1	0.755	0.933	0.936	0.653
	EL2	0.881			
	EL4	0.824			
	EL6	0.84			
	EL7	0.769			
	EL8	0.775			
	EL9	0.727			
	EL10	0.862			
Employee Commitment	EC1	0.832	0.947	0.948	0.592
	EC2	0.799			
	EC3	0.722			
	EC4	0.777			
	EC6	0.776			
	EC7	0.725			
	EC8	0.787			
	EC9	0.742			
	EC10	0.789			
	EC11	0.769			
	EC12	0.756			
	EC13	0.74			
	EC14	0.763			
	EC15	0.832			
Islamic Work Ethic	IWE1	0.748	0.947	0.959	0.575
	IWE2	0.791			
	IWE3	0.734			
	IWE4	0.713			
	IWE5	0.801			
	IWE6	0.794			
	IWE7	0.736			
	IWE8	0.806			
	IWE10	0.763			
	IWE11	0.712			
	IWE12	0.756			
	IWE13	0.725			
	IWE15	0.743			
	IWE16	0.74			
	IWE17	0.804			
Organisational Citizenship Behaviour -Individual	OCB1	0.786	0.897	0.899	0.581
	OCB2	0.789			
	OCB3	0.718			
	OCB4	0.793			
	OCB5	0.767			
	OCB6	0.715			
	OCB7	0.742			
	OCB8	0.784			
Organisational Citizenship Behaviour -Organisational	OCB9	0.698	0.889	0.897	0.602
	OCB10	0.799			
	OCB11	0.787			
	OCB12	0.745			
	OCB13	0.83			
	OCB14	0.761			
	OCB16	0.801			

Internal consistency reliability was assessed using Cronbach’s alpha and composite reliability. All constructs exceeded conventional acceptability thresholds, indicating satisfactory internal consistency. Convergent validity was assessed using the average variance extracted (AVE), and all AVE values exceeded 0.50, indicating that each construct explained more than half of the variance in its indicators (Fornell and Larcker, 1981). Taken together, the reliability and convergent-validity results support the adequacy of the retained measurement model.

All constructs were modelled as reflective, consistent with their conceptualisation as latent attitudinal or behavioural tendencies measured through interchangeable indicators. Discriminant validity was assessed using two complementary procedures. First, the indicator cross-loadings showed that each item loaded most strongly on its intended construct. Second, the heterotrait-monotrait ratio (HTMT) was examined. All HTMT values were below the recommended threshold of 0.90, supporting discriminant validity among the constructs (Henseler et al., 2015). Although the HTMT value between OCBI and OCBO was comparatively high, it remained below the recommended threshold, which is consistent with the expectation that the two forms of citizenship are related but empirically distinguishable (see Table 4).

**Table 4:** Discriminant validity using HTMT – Matrix.

Construct	1	2	3	4	5
1. Employee Commitment (EC)	—				
2. Ethical Leadership (EL)	0.622	—			
3. Islamic Work Ethic (IWE)	0.401	0.25	—		
4. OCB toward individuals (OCBI)	0.672	0.437	0.263	—	
5. OCB toward Organisation (OCBO)	0.692	0.447	0.279	0.851	—

### 4.2 Structural model

The structural model was then assessed to examine the direct, indirect, and conditional relationships among ethical leadership, employee commitment, Islamic Work Ethic, and organisational citizenship behaviour. Ethical leadership was specified as the focal leadership construct, employee commitment as the mediating attitudinal mechanism, and Islamic Work Ethic as the follower-level moderator conditioning the first-stage relationship. Bootstrapping with 5,000 resamples was used to estimate standard errors and significance levels for all hypothesised paths. In addition to the focal paths, direct paths from ethical leadership to OCBI and OCBO were retained to evaluate whether the pattern was more consistent with direct-and-indirect effects or with an indirect-only association in the estimated model.

#### 4.2.1 Hypothesis testing

As reported in Table 5, ethical leadership showed a strong positive association with employee commitment (*β* = 0.542, *t* = 14.906, *p* < 0.001), supporting H1. This finding is consistent with the proposition that employees who perceive their leaders as ethical, fair, and principled report stronger attachment to the organisation.

**Table 5:** Direct, indirect, and conditional effects (Bootstrapping results).

Effect (Hypothesis/Test)	Relationship	*β*	*t*	*p*	Decision
H1	EL → EC	0.54	14.906	<0.001	Supported
H2a	EC → OCBI	0.59	13.482	<0.001	Supported
H2b	EC → OCBO	0.6	13.718	<0.001	Supported
H3a (Mediation)	EL → EC → OCBI	0.32	10.009	<0.001	Supported
H3b (Mediation)	EL → EC → OCBO	0.33	9.934	<0.001	Supported
H4 (Moderation)	EL × IWE → EC	0.24	6.483	<0.001	Supported
H5a (Moderated mediation)	EL × IWE → EC → OCBI	0.14	5.562	<0.001	Supported
H5b (Moderated mediation)	EL × IWE → EC → OCBO	0.14	5.679	<0.001	Supported
Additional test (Direct effect)	EL → OCBI	0.05	1.102	0.135	Not significant
Additional test (Direct effect)	EL → OCBO	0.06	1.178	0.119	Not significant

EL, Ethical Leadership; EC, Employee Commitment; IWE, Islamic Work Ethic; OCBI, OCB toward individuals; OCBO, OCB toward Organisation. Reported statistics are bootstrapped estimates (two-tailed).

Employee commitment, in turn, was positively associated with both citizenship behaviours directed toward individuals (*β* = 0.590, *t* = 13.482, *p* < 0.001) and citizenship behaviour directed toward the organisation (*β* = 0.603, *t* = 13.718, *p* < 0.001), supporting H2a and H2b. These coefficients indicate that commitment is strongly associated with discretionary contribution across both interpersonal and organisational targets.

By contrast, once employee commitment was included in the structural model, the direct path from ethical leadership to OCBI was not statistically significant (*β* = 0.054, *t* = 1.102, *p* = 0.135), nor was the direct path from ethical leadership to OCBO (*β* = 0.061, *t* = 1.178, *p* = 0.119). This pattern suggests that, within the estimated model, the association between ethical leadership and citizenship behaviour is not explained by a remaining direct path once commitment is taken into account. Because the data are cross-sectional, this pattern should be interpreted cautiously as an indirect-only association in the model rather than as definitive evidence of causal full mediation (Rönkkö and Evermann, 2013).

#### 4.2.2 Indirect and interaction effects

The bootstrapped indirect effects provide support for the proposed mediation hypotheses. The indirect association between ethical leadership and OCBI through employee commitment was positive and statistically significant (*β* = 0.320, *t* = 10.009, *p* < 0.001), supporting H3a. Likewise, the indirect association between ethical leadership and OCBO through employee commitment was positive and statistically significant (*β* = 0.327, *t* = 9.934, *p* < 0.001), supporting H3b. These findings indicate that employee commitment functions as a key intervening mechanism linking ethical leadership with both forms of citizenship behaviour.

The interaction between ethical leadership and Islamic Work Ethic also had a significant positive association with employee commitment (*β* = 0.238, *t* = 6.483, *p* < 0.001), supporting H4. This result indicates that the positive association between ethical leadership and commitment is stronger at higher levels of Islamic Work Ethic. In conceptual terms, the findings are consistent with a first-stage moderated mediation model in which follower value orientation shapes the extent to which leadership cues are translated into commitment (Edwards and Lambert, 2007).

Consistent with this moderation result, the conditional indirect association between ethical leadership and OCBI through employee commitment was significant (*β* = 0.140, *t* = 5.562, *p* < 0.001), supporting H5a, and the corresponding conditional indirect association for OCBO was also significant (*β* = 0.144, *t* = 5.679, *p* < 0.001), supporting H5b. Taken together, the results indicate that the indirect association between ethical leadership and citizenship behaviour through commitment becomes stronger as Islamic Work Ethic increases. The overall pattern is therefore consistent with the theorised moderated mediation model.

#### 4.2.3 Robustness check: control variables

To address the possible influence of demographic characteristics, an additional robustness analysis was conducted including gender, age, education, work experience, and managerial status as control variables predicting employee commitment, OCBI, and OCBO. These variables were included because they are commonly reported demographic characteristics in organisational survey research and may plausibly relate to employee attitudes and discretionary behaviour. At the same time, following methodological guidance that control variables should be theoretically justified and reported transparently rather than included automatically, we treated this analysis as a robustness check rather than as the primary model specification (Becker, 2005; Spector and Brannick, 2011; Bernerth and Aguinis, 2016). The inclusion of these controls did not materially change the direction, magnitude, or statistical significance of the hypothesised relationships. Ethical leadership remained positively associated with employee commitment, employee commitment remained positively associated with both OCBI and OCBO, and the ethical leadership × Islamic Work Ethic interaction remained significant. The indirect and conditional indirect effects also remained significant. Accordingly, the results appear robust to the inclusion of demographic controls. For reasons of theoretical parsimony, the main results are reported without controls.

#### 4.2.4 Explanatory power of the structural model

Beyond path significance, the explanatory power of the model was examined using the coefficient of determination (*R*^2^) for each endogenous construct. As shown in Table 6, ethical leadership, Islamic Work Ethic, and their interaction jointly explained 52% of the variance in employee commitment. This indicates substantial explained variance in the focal attitudinal mechanism. Employee commitment, in turn, explained 35% of the variance in OCBI and 38% of the variance in OCBO, indicating meaningful explanatory power for both citizenship outcomes. Given the multifactorial nature of discretionary work behaviour, these values suggest that the model captures a meaningful share of variance in both commitment and citizenship behaviour.

**Table 6:** Explanatory power of the structural model.

Endogenous construct	*R* ^2^	Explanatory power
Employee commitment (EC)	0.52	Substantial
OCB toward individuals (OCBI)	0.35	Moderate
OCB toward Organisation (OCBO)	0.38	Moderate

#### 4.2.5 Effect size (f^2^) analysis

Effect sizes were examined using f^2^ values to assess the relative substantive contribution of each predictor in the structural model. As shown in Table 7, employee commitment displayed strong substantive effects on both OCBI (*f*^2^ = 0.483) and OCBO (*f*^2^ = 0.542), indicating that commitment is the most influential predictor of the two citizenship outcomes in the estimated model. Ethical leadership also showed a strong substantive contribution to employee commitment (*f*^2^ = 0.657), reinforcing its central role in explaining variation in the mediating construct.

**Table 7:** Effect size (f^2^) of structural relationships.

Predictor → Outcome	f^2^	Interpretation
EC → OCBI	0.483	Large
EC → OCBO	0.542	Large
EL → EC	0.657	Large
*(Additional test)* EL → OCBI	0.001	Negligible
*(Additional test)* EL → OCBO	0.001	Negligible
IWE → EC	0.167	Medium
EL × IWE → EC	0.158	Medium

*f*^2^ values of 0.02, 0.15, and 0.35 indicate small, medium, and large effects, respectively (Cohen, 1988; Hair et al., 2022). EL, Ethical leadership; EC, Employee commitment; IWE, Islamic Work Ethic; OCBI, OCB toward individuals; OCBO, OCB toward organisation.

By contrast, the direct paths from ethical leadership to OCBI and OCBO yielded negligible effect sizes (both *f*^2^ = 0.001), which is consistent with the pattern of non-significant direct coefficients reported earlier. Islamic Work Ethic (*f*^2^ = 0.167) and the ethical leadership × Islamic Work Ethic interaction (*f*^2^ = 0.158) both made meaningful contributions to explaining employee commitment, supporting the view that follower ethical orientation conditions how strongly ethical leadership is associated with organisational attachment. Overall, the effect-size pattern reinforces the central empirical pattern of the model: ethical leadership is most strongly associated with citizenship behaviour through employee commitment, and this process is amplified by Islamic Work Ethic.

## 5 Discussion

This study examined how ethical leadership is associated with organisational citizenship behaviour and the conditions under which this association becomes stronger. The findings indicate that ethical leadership is positively associated with employee commitment, and that employee commitment, in turn, is positively associated with both citizenship behaviour directed toward individuals (OCBI) and citizenship behaviour directed toward the organisation (OCBO). Once employee commitment was included in the model, ethical leadership did not show additional direct relationships with either form of citizenship behaviour. In addition, Islamic Work Ethic strengthened the relationship between ethical leadership and employee commitment, as well as the corresponding conditional indirect relationships linking ethical leadership to both forms of citizenship through commitment. Collectively, the findings are consistent with a first-stage moderated mediation model in which ethical leadership is associated with citizenship behaviour primarily through an attitudinal mechanism, and in which this process is stronger when follower values are more closely aligned with the ethical content of leadership.

### 5.1 Ethical leadership and citizenship behaviour: an indirect and contingent relationship

One notable finding is that the direct relationships between ethical leadership and both forms of organisational citizenship behaviour were not statistically significant once employee commitment was modelled, whereas the indirect relationships through commitment were significant. More specifically, the findings indicate that ethical leadership alone did not explain additional variance in OCBI or OCBO once employee commitment was incorporated into the model, suggesting that the attitudinal pathway accounted for the substantive relationship between leadership and discretionary behaviour.

This pattern does not imply that ethical leadership is never directly associated with citizenship behaviour. Rather, it suggests that, within the present model, the relationship is more convincingly explained through an intervening attitudinal process than through a remaining direct path. This interpretation is consistent with broader ethical leadership scholarship showing that leadership effects often operate through distinct mediating mechanisms and that the strength and form of these effects vary across outcomes and conditions (Bedi et al., 2016; Peng and Kim, 2020; Sun et al., 2024).

This interpretation also encourages a more disciplined reading of the relationship between ethical leadership and extra-role behaviour. Rather than treating ethical leadership as a uniform behavioural trigger, the present findings suggest that its association with citizenship behaviour depends on what it changes in employees. This interpretation follows directly from the disappearance of the direct effects once commitment was included, indicating that ethical leadership became relevant for discretionary employee behaviour, primarily through its association with employees’ organisational attachment. In this study, the findings suggest that leader conduct alone is insufficient unless it is associated with stronger employee attachment to the organisation. This provides a more process-oriented account of ethical leadership and is better aligned with the view that leadership effects are often indirect and contingent rather than simple and uniform (Brown and Treviño, 2006; Vigoda-Gadot, 2006; Peng and Kim, 2020).

At the same time, the findings should not be interpreted as evidence that citizenship behaviour was necessarily experienced as fully voluntary or free from pressure. Research on organisational citizenship behaviour has increasingly shown that extra-role behaviour can be beneficial while also carrying strain, expectation, or pressure costs for employees (Bolino et al., 2010; Bolino and Grant, 2016; Organ, 2018; Ling et al., 2025). Because the present study did not directly measure citizenship pressure, compulsory citizenship, or perceived voluntariness, the contribution of the paper is not to resolve that normative issue. Rather, it is to show that the linkage between ethical leadership and citizenship behaviour is more convincingly modelled as a commitment-based and value-contingent process.

### 5.2 Employee commitment as the proximal attitudinal mechanism

The results position employee commitment as the central explanatory mechanism in the model. Ethical leadership was strongly associated with employee commitment, and commitment was, in turn, strongly associated with both OCBI and OCBO. The magnitude and significance of these paths suggest that commitment was not merely an accompanying attitudinal variable, but the primary explanatory mechanism linking leadership perceptions to discretionary contribution. This pattern is theoretically consistent with social exchange reasoning, which suggests that fair, trustworthy, and principled treatment can strengthen employees’ attachment to the organisation and increase their willingness to contribute beyond formal job requirements (Cropanzano and Mitchell, 2005). It is also consistent with longstanding evidence that employee attitudes are among the strongest predictors of organisational citizenship behaviour and related forms of discretionary contribution (Organ and Ryan, 1995).

This mechanism is important because it shifts the theoretical emphasis from leadership as direct behavioural control to leadership as a relational and attitudinal influence. Ethical leadership may matter for citizenship not simply because employees emulate leader conduct, but because such conduct strengthens how employees evaluate and relate to the organisation itself. In this sense, employee commitment provides a more proximal explanation of why ethical leadership is associated with discretionary contribution than models that assume a direct leadership-to-behaviour relationship.

The finding that commitment is associated with both OCBI and OCBO also strengthens the explanatory coherence of the model. Although the two forms of citizenship differ in target, both appear to be linked to a common attitudinal foundation. This is broadly in line with research showing that organisational citizenship behaviour directed at individuals and at the organisation can be meaningfully distinguished while still sharing common attitudinal antecedents (Lee and Allen, 2002; Organ, 2018). The present study therefore adds precision by showing that a commitment-based pathway is relevant to both interpersonal and organisation-directed forms of discretionary behaviour.

### 5.3 Islamic work ethic as a follower-level boundary condition

A further contribution of the study lies in showing that the ethical leadership–commitment relationship is not uniform across employees. Islamic Work Ethic strengthened the relationship between ethical leadership and commitment, and it also strengthened the corresponding indirect relationships between ethical leadership and both forms of citizenship behaviour. This suggests that follower values matter in shaping how leadership cues are interpreted and translated into work attitudes. These findings suggest that follower values shape the extent to which ethical leadership cues are translated into organisational attachment and subsequent discretionary behaviour. This is consistent with broader work on value congruence, which argues that employees’ responses to organisational signals depend partly on the extent to which those signals resonate with their own value frameworks (Edwards and Cable, 2009). It is also consistent with ethical leadership research showing that follower moral characteristics shape the extent to which employees attend to, interpret, and respond to the moral content of leadership (van Gils et al., 2015). In the present study, Islamic Work Ethic appears to function as one such interpretive lens. When employees hold a stronger ethical orientation toward work, the behavioural signals associated with ethical leadership may be more likely to be perceived as normatively meaningful and personally relevant, thereby strengthening their association with commitment.

Importantly, the role of Islamic Work Ethic in this model should not be reduced to a mere background cultural variable. The findings suggest a more active theoretical role: follower ethical orientation helps determine how strongly ethical leadership is associated with organisational attachment. This strengthens the case for moving beyond leader-centric explanations of ethical leadership and for incorporating follower interpretive processes more explicitly in future leadership research.

### 5.4 Theoretical contributions

The theoretical contributions emerge directly from the combined pattern of non-significant direct effects, significant indirect effects, and significant moderation and moderated mediation relationships observed in the structural model. The study makes three main theoretical contributions. First, it contributes to ethical leadership research by showing that the relationship with organisational citizenship behaviour is more convincingly explained as an indirect, commitment-based process than as a straightforward direct behavioural effect. In this way, the study refines prior ethical leadership research by identifying employee commitment as a proximal attitudinal mechanism through which ethical leadership becomes associated with discretionary contribution. Second, the study shows that this process is contingent on follower value orientation. By demonstrating the moderating role of Islamic Work Ethic, it extends ethical leadership research beyond predominantly leader-centric explanations and highlights the importance of follower interpretive frameworks in shaping how leadership cues are translated into work attitudes and behaviour. Third, by distinguishing between OCBI and OCBO, the study offers a more conceptually precise account of citizenship behaviour and shows that the proposed commitment-based pathway is relevant across both interpersonal and organisation-directed forms of extra-role contribution.

The study also contributes contextually. The evidence comes from public-sector and large organisational settings in Qatar, and prior meta-analytic evidence suggests that ethical leadership relationships can vary across settings and may be stronger in public-sector samples (Bedi et al., 2016). The present findings therefore add useful contextual depth to ethical leadership research while still speaking to broader theoretical debates about mechanism and contingency.

### 5.5 Practical implications

The findings carry several practical implications. First, organisations should be cautious about treating ethical leadership as a direct lever for increasing extra-role behaviour. The present results suggest that ethical leadership is more likely to be associated with citizenship behaviour when it strengthens employees’ attachment to the organisation. In practical terms, this means that leadership development initiatives should not focus only on visible ethical conduct, but also on the relational conditions through which such conduct becomes meaningful to employees, including fairness, trust, consistency, and credible role modelling (Brown and Treviño, 2006).

Second, the results suggest that organisations should pay greater attention to follower interpretation. The same leadership behaviour may not be interpreted identically by all employees, and leadership effects may be stronger when leader conduct resonates with employees’ ethical orientation. In value-rich organisational settings, leaders may therefore need to communicate ethical expectations in ways that are not only procedurally fair but also normatively intelligible to employees.

Third, the practical goal should not involve pressuring employees into citizenship behaviour. Research on citizenship pressure warns that extra-role contribution can become socially expected in ways that create strain or ambiguity for employees (Bolino et al., 2010; Bolino and Grant, 2016). The practical implication of the present study is therefore not that managers should demand more citizenship, but that they should create relational and ethical conditions under which employees are more likely to contribute voluntarily and sustainably.

### 5.6 Limitations and directions for future research

The study should be interpreted in light of several limitations. First, the data are cross-sectional, which limits strong claims about temporal ordering. Although the hypothesised model is theoretically grounded, the results are best interpreted as theory-consistent associations rather than definitive evidence of causal sequence. Future research would benefit from time-lagged or longitudinal designs that permit stronger inference about how ethical leadership, commitment, and citizenship behaviour unfold over time.

Second, the study relies on self-reported data collected from a single source. Although procedural remedies and diagnostic tests were applied, common method bias cannot be ruled out completely in single-source survey research (Podsakoff et al., 2003; Podsakoff et al., 2012).

Third, the study did not directly measure citizenship pressure, compulsory citizenship, or perceived voluntariness. This remains important because contemporary OCB research increasingly recognises that extra-role behaviour can be both valuable and potentially burdensome (Bolino et al., 2010; Bolino and Grant, 2016; Organ, 2018). Future studies should therefore examine whether ethical leadership is associated not only with higher levels of citizenship behaviour, but also with lower levels of felt pressure or greater perceived voluntariness in performing such behaviour (Li et al., 2025).

Finally, although Islamic Work Ethic performed as a meaningful moderator in this study, future research should examine whether similar moderating patterns emerge for other follower value orientations, such as professional ethical commitment, public service values, or broader moral identity constructs. Comparative work across different normative contexts would help clarify which aspects of the present model are context-specific and which reflect more general follower-level contingencies in ethical leadership processes.

## 6 Conclusion

This study examined the attitudinal and value-contingent processes through which ethical leadership is associated with organisational citizenship behaviour. The findings indicate that ethical leadership is positively associated with employee commitment, that employee commitment is positively associated with both citizenship behaviour directed toward individuals and toward the organisation, and that Islamic Work Ethic strengthens both the ethical leadership–commitment relationship and the corresponding conditional indirect relationships with both forms of citizenship behaviour.

The contribution of the study lies in showing that the relationship between ethical leadership and citizenship behaviour is more convincingly explained as a commitment-based and value-contingent process than as a straightforward direct behavioural effect.

In this respect, the findings refine existing work on ethical leadership by identifying employee commitment as a proximal attitudinal mechanism and Islamic Work Ethic as a meaningful follower-level boundary condition.

Nevertheless, the study does not claim that ethical leadership directly produces fully voluntary or morally unambiguous citizenship behaviour. Because the design is cross-sectional and self-reported, and because perceived pressure or compulsory citizenship was not directly measured, the findings are best interpreted with appropriate caution. Even so, they provide a more theoretically disciplined account of how ethical leadership is associated with discretionary contribution in a public-sector Islamic-context setting.

Overall, the study advances research on ethical leadership and organisational behaviour by showing that leadership influence depends not only on whether leaders are perceived as ethical, but also on whether such leadership strengthens organisational attachment and resonates with employees’ value orientations. This offers a clearer basis for future research on leadership, employee attitudes, and discretionary work behaviour.

## Data Availability

The raw data supporting the conclusions of this article will be made available by the authors, without undue reservation.
